# What Is New in Genetics of Congenital Heart Defects?

**DOI:** 10.3389/fped.2016.00120

**Published:** 2016-12-01

**Authors:** Maria Cristina Digilio, Bruno Marino

**Affiliations:** ^1^Medical Genetics, Bambino Gesù Pediatric Hospital, Rome, Italy; ^2^Pediatric Cardiology, Department of Pediatrics, Sapienza University, Rome, Italy

**Keywords:** genetics, congenital heart defect, syndrome, chromosome, genetic counseling

## Abstract

Epidemiological studies, clinical observations, and advances in molecular genetics are contributing to the understanding of the etiology of congenital heart defects (CHDs). Several phenotype–genotype correlation studies have suggested that specific morphogenetic mechanisms put in motion by genes can result in a specific cardiac phenotype. The use of new technologies has increased the possibility of identification of new genes and chromosomal loci in syndromic and non-syndromic CHDs. There are a number of methods available for genetic research studies of CHDs, including cytogenetic analysis, linkage and association studies, copy number variation (CNV) and DNA micro-array analysis, and whole exome sequencing. The altered dosage of contiguous genes included inside CNVs can produce new syndromic CHDs, so that several different new genomic conditions have been identified. These include duplication 22q11.2 syndrome, distal 22q11.2 deletion syndrome, deletion and duplication 1q21.1, and deletion 1p36 syndrome. Molecular techniques such as whole exome sequencing have lead to the identification of new genes for monogenic syndromes with CHD, as for example in Adams–Oliver, Noonan, and Kabuki syndrome. The variable expressivity and reduced penetrance of CHDs in genetic syndromes is likely influenced by genetic factors, and several studies have been performed showing the involvement of modifier genes. It is not easy to define precisely the genetic defects underlying non-syndromic CHDs, due to the genetic and clinical heterogeneity of these malformations. Recent experimental studies have identified multiple CNVs contributing to non-syndromic CHD. The number of identified genes for non-syndromic CHDs is at this time limited, and each of the identified genes has been shown to be implicated only in a small proportion of CHD. The application of new technologies to specific cases of CHD and pedigrees with familial recurrence and filtering genes mapping in CNV regions can probably in the future add knowledge about new genes for non-syndromic CHDs.

## Introduction

Epidemiological studies, clinical observations, and advances in molecular genetics are contributing to the understanding of the etiology of congenital heart defects (CHDs). The majority of CHDs are occurring as isolated malformations, while approximately 25–30% of them are associated with extracardiac anomalies, in the setting of large or submicroscopic chromosomal anomalies, Mendelian disorders, and malformation associations (1–3). Some types of CHD, such as atrioventricular canal defect (AVCD) and interrupted aortic arch (IAA), are more frequently found in association with genetic syndromes, whereas other types are prevalently isolated defects (tricuspid atresia, transposition of the great arteries, and pulmonary atresia). Several phenotype–genotype correlation studies have suggested that specific morphogenetic mechanisms put in motion by genes can result in a specific cardiac phenotype (4). Clinical implications are resulting from these studies, since distinct cardiac anatomic subtypes may help in suggesting accurate diagnoses, which can be confirmed by molecular testing.

It should be considered that it is not easy to define precisely the genetic defects underlying CHDs, particularly non-syndromic types, due to the genetic and clinical heterogeneity of these malformations. Multiple parallel approaches are needed for the exploration of the potential loci etiologically related to CHDs. Rapid advances in genetic technologies have substantially improved the possibility to detect new genes and chromosomal regions for CHDs. There are a number of methods available for genetic research studies of CHDs, including cytogenetic analysis, linkage and association studies, copy number variation (CNV) and DNA micro-array analysis, and whole exome sequencing. The use of new technologies has increased the possibility of identification of new genes and chromosomal loci in syndromic and non-syndromic CHDs.

## Syndromic CHDs

The 30% of patients with syndromic CHD can be affected by chromosomal anomalies, Mendelian syndromes, and non-Mendelian associations.

Classic chromosome anomalies detectable by normal standard karyotype include trisomy 21 (Down syndrome), trisomy 13 (Patau syndrome), and trisomy 18 (Edwards syndrome), monosomy X (Turner syndrome), deletion 8p23.1, and terminal deletion 4p. Each chromosomal anomaly is preferentially associated with specific types of CHDs, as occurring for AVCD and Down syndrome or aortic coarctation and Turner syndrome.

The more frequent chromosomal syndromes linked to submicroscopic anomalies detectable by Fluorescent *in situ* hybridization analysis (FISH) or arrayCGH are the 22q11.2 deletion (DiGeorge/velocardiofacial) with conotruncal heart defects and Williams syndromes with supravalvular aortic stenosis.

Monogenic syndromes characteristically associated with CHD are represented by Noonan syndrome and other RASopathies, Alagille, Kabuki, Ellis–van Creveld, Holt–Oram, CHARGE, Marfan, and Cornelia de Lange syndromes. Malformation associations are including VACTERL, Goldenhar (oculo-auriculo-vertebral spectrum), and Cantrell.

## New Chromosomal Syndromes with CHD

The development of molecular technologies is leading to the identification of new subchromosomal changes in genome structure, known as CNVs. These are deletions or amplifications of DNA segments arising from inappropriate chromosomal recombination, due to flanking region-specific repeat sequences or from highly homologous genes that misalign during meiosis. CNVs can be identified using arrayCGH or genomic microarrays that assess single-nucleotide polymorphisms (SNPs). The altered dosage of contiguous genes included inside CNVs can produce new syndromic CHDs, so that several different new genomic conditions have been identified.

### Duplication 22q11.2 Syndrome

The duplication 22q11.2 syndrome is the reciprocal product of the 3-Mb chromosomal region deleted in DiGeorge/velocardiofacial syndrome (between LCRA and LCRD) (5, 6). The most frequently reported features are mental retardation, learning difficulties, ADHD, growth retardation, and facial anomalies. CHDs, visual and hearing impairment, seizures, microcephaly, ptosis, and urogenital abnormalities have also been reported. The prevalence of CHD in duplication 22q11.2 is lower in comparison to that of deletion 22q11.2, and the spectrum of CHDs wider, including defects belonging to different pathogenetic pathways (septal defects, conotruncal heart defect, and left-sided obstructive lesions). The molecular basis leading to CHD is to be elucidated, considering the overexpression of the TBX1 gene and the possible interaction with other genes inside and outside the 22q11.2 chromosomal region.

### Distal 22q11.2 Deletion Syndrome

The distal 22q11.2 deletion syndrome is a new genomic disorder mapping at the telomeric end of the common DiGeorge/22q11.2 deleted region (7). Clinical features of the 22q11.2 distal deletion syndrome include developmental delay, facial anomalies, low birth weight, skeletal anomalies, and CHDs. Anatomic types of CHD include ventricular septal defects, truncus arteriosus, double outlet right ventricle, aortic anomalies, and left ventricular non-compaction (8). Particularly, left ventricular non-compaction with aortic valve anomalies has been recently reported as useful diagnostic marker for the syndrome (9). Genes functionally interacting with TBX1, but mapping distal to this gene, such as CRKL and ERK2/MAPK1, have been proposed as candidate to CHD in this syndrome, in particular haploinsufficiency of MAPK1 gene, located between LCR22-4 and LCR22-5 (8). It has been observed that smaller deletions seem to be associated with the more complex CHDs, particularly those involving LCR22-4 to LCR22-5 and LCR22-4 to LCR22-6.

### Deletion and Duplication 1q21.1

Microdeletion 1q21.1 (del 1q21.1) and the reciprocal microduplication 1q21.1 (dup 1q21.1) are genomic disorders characterized by developmental delay, dysmorphic features, and congenital malformations (10). CHD is a major feature of deletion 1q21.1 and has been occasionally reported in duplication 1q21.1 (10–12). The anatomic types of CHD diagnosed in deletion 1q21.1 are heterogeneous, including mainly left-sided obstructions (40%), as aortic coarctation, bicuspid aortic valve, and subaortic stenosis, but also septal defects (27%) and conotruncal anomalies (20%). Our group has reported a family segregating deletion 1q21.1 in three members, two of whom had CHD, including syndromic atrial septal defect, pulmonary valve stenosis, and muscular ventricular septal defects, and the maternal uncle of this proband with non-syndromic pulmonary valve stenosis (12). This finding prompted the investigation of the role of recurrent rearrangements of chromosome 1q21.1 region in the pathogenesis of pulmonary valve stenosis. The results of this study were that duplication 1q21.1 was detected in a single sporadic non-syndromic patient. Soemedi et al. (13) studied recurrent rearrangements of chromosome 1q21.1 in 2436 patients with CHD, demonstrating that duplication 1q21.1 was more common in cases with tetralogy of Fallot, while deletion 1q21.1 was associated with CHDs different from tetralogy of Fallot.

Inside the 1q21.1 chromosomal region are mapping genes important for CHD development. At least two of these genes, AMP-activated protein kinase (AMPK) and GJA5 (Cx40), are expressed in the cardiac tissue. Mutations in AMPK are occurring in a subset of subjects affected by familial hypertrophic cardiomyopathy (14). Cx40 gene imbalances could account for structural cardiac defects in del 1q21.1. In fact, Cx40 haploinsufficient mice can have different types of CHD, including ventricular septal defect, tetralogy of Fallot, and aortic arch abnormalities (15). Recently, GJA5 gene has been demonstrated to be a susceptibility gene for non-syndromic tetralogy of Fallot in humans (13, 16).

### Deletion 1p36 Syndrome

Deletion 1p36 is one of the most common genomic disorders, and the second most common deletion syndrome, characterized by intellectual disability, epilepsy, CHD, and characteristic dysmorphic facial features (17). CHD is diagnosed in about 50% of these patients, which includes cardiomyopathy and a high prevalence of left ventricular non-compaction (17, 18). This type of cardiomyopathy is characterized by prominent left ventricular trabeculae and deep intratrabecular recesses. Associated extracardiac anomalies occur in 14–66% of the patients in different series, in the setting of syndromic, metabolic, and neuromuscular disorders (19). Interestingly, the transcription factor PRDM16, mapping inside the critical region of 1p36 syndrome, has been established to be linked to a proportion of non-syndromic left ventricular non-compaction patients (20).

A specific association has been noted also between Ebstein anomaly, an uncommon CHD characterized by downward displacement of the tricuspid valve into the right ventricle, and deletion 1p36 syndrome (21).

## New Genes for Monogenic Syndromes with CHD

### Adams–Oliver Syndrome

Adams–Oliver syndrome is a rare developmental disorder characterized by the combination of aplasia cutis congenita of the scalp vertex and terminal transverse limb defects (22). In addition, vascular anomalies and CHD are frequently observed. CHDs have been estimated to be present in 20% of individuals with Adams–Oliver syndrome, including various anatomic types with a specific association with left-sided obstructive lesions (23). The syndrome was known to be etiologically heterogeneous, since mutations in four different genes (ARHGAP31, RBPJ, DOCK6, and EOGT) have been detected in about 10% of individuals with this syndrome, particularly without cardiac involvement (22). Recently, heterozygous variants in NOTCH1 gene, belonging to the Notch signaling pathway, have been shown to be related to Adams–Oliver syndrome with CHD (24, 25). It has been proposed that the limb and scalp defects might also be due to a vasculopathy in NOTCH1-related AOS. Interestingly, germline *NOTCH1* variants affecting diverse domains of the protein can be associated with autosomal dominant non-syndromic CHDs affecting the left ventricular outflow tract, most commonly bicuspid aortic valve, with an additional frequent feature of adult-onset precocious aortic valve calcification (26).

### Kabuki Syndrome

Kabuki syndrome is a genetic disease causing developmental delay and congenital malformations with specific facial features. Left-sided cardiac obstructive lesions are known to be the first type of CHDs for frequency in these patients, although septal and conotruncal defects can also be detected (27). The involvement of X chromosome has been previously suspected in order to justify the high prevalence of left-sided defects (27). Since the identification of MLL2 gene mutations as the primary cause of this syndrome (28), the involvement of the second gene has been discovered (29). In fact, *de novo* partial or complete deletions of an X chromosome gene, KDM6A, which encodes a histone demethylase that interacts with MLL2, so as heterozygous gene mutations of the same gene have been diagnosed in patients with Kabuki phenotype, identifying KDM6A as another cause of Kabuki syndrome.

### Noonan Syndrome

Noonan syndrome and related disorders, including Leopard, cardiofaciocutaneous, and Costello syndromes (the so-called RASopathies), are causally linked to germline mutations in a number of genes coding transducers and modulatory proteins participating in the RAS–MAP kinase (MAPK) signaling pathway (30). Clinical features include dysmorphic features, CHDs, postnatal growth retardation, ectodermal and skeletal defects, and variable cognitive deficits (30). CHDs occur in approximately 60–90% of patients affected by one of these RASopathies, depending on the mutated genes. Pulmonary valve stenosis and hypertrophic cardiomyopathy are the most common defects displaying a distinct association with the RASopathies (31). Noonan syndrome and other RASopathies are genetically heterogeneous, and mutations in the PTPN11, SOS1, KRAS, RAF1, BRAF, SHOC2, MEK1, and MEK2 genes have been documented (30). A new gene for RASopathies is being discovered every 6 months in the last years, and after the identification of the additional genes NRAS (32), CBL (33), RIT1 (34), and LZTR1 (35) the percentage of molecularly confirmed Noonan syndrome patients is 90%. CHDs associated with mutations is these new genes are the classic ones. The prevalence of CHDs in patients with RIT1 gene is particularly high (90%) (36).

In addition, it has been confirmed by molecular diagnosis that AVCD is a part of the phenotypic spectrum of CHDs found in patients with RASopathies, in particular those caused by PTPN11 and RAF1 gene mutations, as the third most common CHD (37). Partial atrioventricular canal associated with left-sided obstructions or pulmonary valve stenosis or hypertrophic cardiomyopathy should be regarded as markers for RASopathies.

## Modifier Genes for Syndromic CHDs

The etiology of intrafamilial phenotypical variability of genetic syndromes is currently not known. The variable expressivity and reduced penetrance of CHDs is likely influenced by genetic factors. In deletion 22q11.2 (DiGeorge/velocardiofacial) syndrome, it has been suspected that genetic variants lying outside of the 22q11.2 region are influencing the development of CHD. CNVs have been extensively studied. Recent results have shown that CNVs of the glucose transporter gene *SLC2A3*, particularly duplications, can increase the risk to born with a CHD (38). Murine *SLC2A3* is expressed in the pharyngeal apparatus and cardiac outflow tract during cardiac morphogenesis. In humans, individuals with a duplication of *SLC2A3* in the absence of deletion 22q11.2 do not present with CHDs, and the *SLC2A3* is often transmitted from a parent with normal heart and not carrying deletion 22q11.2, indicating that both mutations may be required for the manifestation of a CHD. This finding supports a possible “two-hit” model. In fact, it appears likely that there may be an epistatic interaction between the *SLC2A3* duplication and dosage sensitive genes in the 22q11.2 deleted region that increase the likelihood of a structural cardiac defect.

## Non-Syndromic CHDs

It is thought that the etiology of the great majority of non-syndromic CHDs can be explained by a “multifactorial” model of inheritance (39). This mechanism of inheritance is reconducted to a genetic predisposition interacting with an environmental trigger. The genetic predisposition is presumed to by polygenic, due to the small additive effects of many genes. However, a single locus or a small number of loci are also considered to be implicated.

## CNVs and Non-Syndromic CHD

Recent experimental studies have identified multiple CNVs contributing to non-syndromic CHD. Pathogenetic *de novo* CNVs have been reported in patients with tetralogy of Fallot, AVCD, and left-sided lesions (40–44). According to the results of these studies, it has been estimated that 5–10% of sporadic non-syndromic CHD can be due to a rare CNV. Some CNVs are mapping in chromosomal regions previously known to contain genes pathogenetically related to CHDs. In fact, recurrent CNVs included chromosome 8p23.1 including the *GATA4* gene or chromosomes 20p12.2 and 9q34.3 where two genes for Alagille syndrome, *JAG1*, and *NOTCH1* are included. The distinction between pathogenetic CNVs and benign polymorphic variants is not always clear. Filtering genes mapping in CNV regions can probably in the future add knowledge about new genes for non-syndromic CHDs.

## Monogenic Inheritance in Non-Syndromic CHDs

Most cases of CHD occur sporadically without a strong family history. Nevertheless, Mendelian monogenic transmission has also been evidenced for some types of CHDs, including atrial septal defects, AVCD, and left-sided obstructive lesions. Monogenic inheritance has been demonstrated particularly in pedigrees with a clear familial recurrence of the defect. Nevertheless, the number of identified genes for non-syndromic CHDs is at this time limited, and each of the identified genes has been shown to be implicated only in a small proportion of CHD (45–51).

Low penetrance has been demonstrated in some pedigrees, when a mutation in a CHD gene in an affected proband is segregating from an unaffected parent (52).

Additive effect of multiple genes can also be considered in some families (53). In fact, it has been observed that mutations in different genes segregating in different manner in relatives can be causally related to CHD, which argues for an oligogenic complex mode of inheritance.

## Clinical Implications

Early and precise genetic diagnosis of patients with CHD is useful for clinical management of children and genetic counseling for families. In fact, possible associated extracardiac anomalies can be monitored and treated. The patients can be followed accordingly to multidisciplinary protocols and guidelines specific for the syndrome (54, 55).

In regard to cardiac risk factors is emerging that specific perioperative protocols may reduce the mortality and morbidity of CHDs in syndromic patients (56). Surgical prognosis of CHDs in some specific syndromes has been studied. Results are showing that surgical risks for complete and partial atrioventricular canal in Down syndrome are not increased, with the exception of the risk for pulmonary hypertension (56, 57). In regard to conotruncal heart defects, microdeletion 22q11.2 syndrome is not a risk factor, and increased surgical mortality is described only for patients with pulmonary atresia with ventricular septal defect (58). Other complex syndromes, such as VACTERL association, can have a negative impact on the surgical prognosis (58).

## Conclusion

Multiple parallel diagnostic approaches are currently used for the identification of the potential loci etiologically related to CHDs, and recent advances in genetic technologies have improved the possibility to detect new genes and chromosomal regions for CHDs. With these methods, several causes of syndromic and isolated CHDs have been discovered, although much work still remains to be done, particularly in respect to non-syndromic CHD, since knowledge of their underlying genetic mechanisms is at present rather limited, due to the multifactorial and complex mode of inheritance.

## Author Contributions

The authors performed the critical review of the manuscript.

## Conflict of Interest Statement

The authors declare that the research was conducted in the absence of any commercial or financial relationships that could be construed as a potential conflict of interest.
